# PSMC5 Promotes Proliferation and Metastasis of Colorectal Cancer by Activating Epithelial–Mesenchymal Transition Signaling and Modulating Immune Infiltrating Cells

**DOI:** 10.3389/fcell.2021.657917

**Published:** 2021-07-16

**Authors:** Zirui He, Xiao Yang, Ling Huang, Leqi Zhou, Sen Zhang, Jing Sun, Minhua Zheng, Junjun Ma, Bo Feng, Lu Zang

**Affiliations:** ^1^Department of General Surgery, Ruijin Hospital, Shanghai Jiao Tong University School of Medicine, Shanghai, China; ^2^Shanghai Minimally Invasive Surgery Center, Ruijin Hospital, Shanghai Jiao Tong University School of Medicine, Shanghai, China

**Keywords:** colorectal cancer, metastasis, tumor infiltrated immune cells, PSMC5, m^6^A

## Abstract

We designed the present study to access the roles and mechanisms of PSMC5 in colorectal cancer (CRC). Transcriptomic and clinical data from public datasets and our center were retrospectively analyzed. Functional assays were performed to investigate the effects of PSMC5 on CRC cells. The results showed that PSMC5 was significantly higher in cancer than normal tissues. Moreover, patients with higher expression of PSMC5 showed poorer prognosis. Silencing of PSMC5 dramatically suppressed the proliferation and invasion of CRC cells, while overexpression led to the opposite. In addition, we screened downstream targets and found that PSMC5 regulates multiple pathways including epithelial–mesenchymal transition, hypoxia, and immune response. Consistently, we found that PSMC5 was negatively correlated with levels of CD8 + T cells and B cells while promoting infiltration of macrophages and neutrophils. Collectively, these findings suggested that PSMC5 was a promising biomarker and target for immune therapy for CRC.

## Introduction

Colorectal cancer (CRC) is known as one of most common digestive malignancies. Despite the tremendous improvements in surgical techniques, adjuvant chemotherapy, and immune therapy, the prognosis of CRC still remains grim (Siegel et al., 2020). Therefore, the need to understand the molecular mechanisms and identify a novel treatment target for CRC treatment is still urgent. Multiple proteins and signaling pathways have been proved to contribute to the formation and progression of CRC. However, the roles of proteasome in CRC remain incompletely clarified (Mani and Gelmann, 2005).

The 26S proteasome is a proteinase complex containing a 20S core particle and a group of 19S regulatory proteins (Hough et al., 1986). PSMC5 (proteasome 26S subunit, ATPase 5) is one of the regulatory components that recognize and transfer ubiquitinated proteins for degradation by the 26S proteasome (Rechsteiner et al., 1993). In addition to the regulation in proteasome functions, accumulating evidence indicated that PSMC5 is directly involved in transcriptional regulation through its interaction with transcriptionally active promoters and the recruitment of co-activators (Zhu et al., 2007). For instance, in the process of adenoviral infection, PSMC5 can be recruited to E1A, a viral transcription factor, and can enhance transcription of downstream targets (Rasti et al., 2006). PSMC5 also can associate with the p53 transcription factor and is recruited to p53 responsive p21 promoters in a manner that correlates with p53 recruitment. Nevertheless, the roles and functions of PSMC5 in tumor, especially in CRC, have not been fully investigated.

In the current study, we found that the expression of PSMC5 was significantly up-regulated in the CRC tissues compared with paired normal tissues and was associated with long-term survival of CRC patients. Loss of PSMC5 could inhibit CRC cell proliferation and colony formation both *in vivo* and *in vitro*. Further study showed that loss of PSMC5 inhibits the migration and invasion of CRC cells. The molecular mechanisms by which PSMC5 regulates CRC cells include the activation of epithelial–mesenchymal transition (EMT) and alteration of immune infiltrates in the tumor microenvironment (TME). Moreover, PSMC5 was associated with response of immune therapy for several types of cancer such as melanoma, non-small cell lung cancer, and urothelial cancer. Taken together, our study suggested that PSMC5 may act as a potential target for CRC treatment.

## Materials and Methods

### Tissue Specimen

All the CRC tissues were obtained from CRC patients who underwent surgical resection in Ruijin Hospital and were diagnosed pathologically by two pathologists. All participants gave written, informed consent, and no participants had received any medication prior to sample collection under the guidelines of Ethics Committee of Ruijin Hospital. The pathological stage was determined according to the criteria of the Union for International Cancer Control. The tumor tissues and paired normal colonic tissues located approximately 10 cm to the distal edge of the tumor were stored in liquid nitrogen for further analysis. The patients were followed up for 90 months or until death. The time of death and recurrence of tumor were recorded under the approval of the Ethics Committee of Ruijin Hospital.

### Cell Lines

The HEK293T, HCT116, and RKO cell lines were previously purchased from American Type Culture Collection (Manassas, VA, United States) and kept in our laboratory. The HEK293T cells were cultured in high-glucose Dulbecco’s modified Eagle’s medium with 10% fetal bovine serum (FBS) at 37°C; HCT116 and RKO cells were maintained in McCoy’s 5A medium (HyClone, Logan, UT, United States) with 10% FBS under a humidified atmosphere at 37°C with CO_2_.

### Establishment of shPSMC5 Colorectal Cancer Cells

To establish the shPSMC5 cells, the short hairpin RNA (shRNA) sequences specifically targeting PSMC5 (5′-AGATTCATTCTCGGAAGAT-3′) was inserted into the GV115 vector. The plasmids were transfected into HEK293T cells using a Lipofectamine 2000 transfection reagent. After 48–72 h, the culture medium was collected, filtered, and then used to infect HCT116 and RKO cells. shPSMC5 cells were obtained by antibiotic selection (puromycin 6 μg/ml).

### Public Datasets

Transcriptome and clinical data of colon adenocarcinoma (COAD) from The Cancer Genome Atlas (TCGA) were downloaded from Xena datahub. The edgeR and limma packages from the Bioconductor project were used for analysis of RNA-seq data. Raw count data were normalized using TMM implemented in edgeR and then were transformed by voom in limma. The expression of PSMC5 in multiple cancer cell lines was analyzed in the datahub of Cancer Cell Line Encyclopedia.

### Cell Growth and MTT Proliferation Assay

For cell growth analysis, the cells were cultured in 96-well plates at an initial density of 2 × 10^3^ cells/well. Each group had three wells (10 μl/well) and was incubated at 37°C in an atmosphere of 5% CO_2_. Fluorescence photomicrographs were captured, and cells with green fluorescence were measured by a Celigo Image Cytometer (Nexcelom Bioscience, Lawrence, MA, United States). Cell growth curves were generated for a time course of 5 days. MTT [3-(4,5-dimethylthiazol-2-yl)-2,5-diphenyltetrazolium bromide] (Genview Scientific Inc., Galveston, TX, United States) was used to measure cell viability. The infected cells (2 × 10^3^ cells/well) were collected and reseeded in 96-well plates. From the first to fifth day, a total of 20 μl of MTT solution (5 mg/ml) was added to the cells. Subsequently, the supernatants were removed, 100 μl of DMSO was added to each well, and the plates were oscillated for 3 min. Finally, the optical density at 490 nm was measured with a microplate reader.

### Immunoblot

Cells were washed with cold phosphate-buffered saline and then lysed with lysis buffer (10 mM of Tris–HCl, 1% Triton X-100, and 150 mM of NaCl) containing protease and phosphatase inhibitor cocktail (Roche, Indianapolis, IN, United States). Insoluble materials were removed by centrifugation at 15,000 *g* for 15 min at 4°C. The concentration of the collected protein was determined by bicinchoninic acid assay (Thermo Fisher Scientific, Waltham, MA, United States). Equal amounts of protein were separated using 10% sodium dodecyl sulfate–polyacrylamide gel electrophoresis, transferred to polyvinylidene difluoride membrane, blocked with 5% non-fat milk at room temperature for 1 h, and immunoblotted with primary antibodies: PSMC5 (Abcam, Cambridge, United Kingdom), GAPDH antibody (Santa Cruz Biotechnology, Dallas, TX, United States), Twist (Abcam), Snail (Cell Signaling Technology, Danvers, MA, United States), Slug (Cell Signaling Technology), and VIM (Cell Signaling Technology). Immunoreactive bands were visualized using an enhanced chemiluminescence kit (Amersham Biosciences, Piscataway, NJ, United States). All experiments were repeated for three times independently.

### Immunohistochemical Staining

The tumor and paired normal tissue sections (4 μm) were deparaffinized, dehydrated, and then treated by 3% H_2_O_2_ at room temperature for 10 min to block endogenous peroxidase activity. Next, the tissue sections were incubated with citrate buffer for the retrieval of the antigen. Then the tissues were blocked with 3% bovine serum albumin at room temperature for 30 min, followed by incubation with PSMC5 antibody (1:100) at 4°C overnight. Finally, the tissue slides were counterstained with hematoxylin and eosin (HE). The expression of PSMC5 was scored and categorized as 0, +1, +2, or +3 according to the intensity of staining according to the reference and was analyzed by Fisher’s exact test.

### Colony Formation Assay

One thousand cells were seeded in six-well plates, and fresh medium was added every 3 days. The cells were cultured for about 15 days, until visible colonies were observed. Then the medium was discarded, and the colonies were fixed with 4% paraformaldehyde and stained with 0.1% crystal violet. The efficiency of colony formation was estimated by the number of colonies.

### Migration and Invasion Assay

The cell migration and invasion assays were performed using transwell chambers (Corning, New York, NY, United States) with 24-well plates. The migration assays were conducted using transwell chambers precoated with 1% collagen I, while the invasion assays used Matrigel-coated (BD Biosciences, San Jose, CA, United States) chambers; 2 × 10^5^ HCT116 cells and RKO cells were suspended in 500 ml of serum-free medium and seeded into the upper chamber; and medium with 10% FBS was added into the lower chamber. After incubation for 24 h for the migration assay and 48 h for the invasion assay, cells were fixed with 4% paraformaldehyde at room temperature for 30 min and then stained with 0.1% crystal violet at 37°C for 1 h. The stained cells were counted in three fields with random choice.

### Caspase-3/7 Assay

Caspase-3/7 are central effector caspases in apoptosis and are usually used to measure apoptotic activities. RKO and HCT116 cells were first transfected with the PSMC5 shRNA and control. Next, 1 × 10^4^ infected cells/well were seeded in a 96−well plate. Caspase−Glo 3/7 reagent (100 μl, G8091; Promega, Madison, WI, United States) was added to each well, the plate was shaken for 30 min constantly, and incubation was carried out at ambient temperature for 2 h. The luminescence signal was detected with an M2009PR (Tecan Infinite, Tecan Trading AG, Männedorf, Switzerland) plate reader.

### Mouse Xenografts

Colorectal cancer cells (1 × 10^6^ cells) were subcutaneously injected into 4-week-old male nude mice. After tumors were detected by palpation, tumor nodules were measured every 7 days, and the mice were euthanized 4 weeks after injection. Tumors were weighed and fixed by formalin. All steps were performed according to the Guide for the Care and Use Laboratory Animals of Ruijin Hospital, Shanghai Jiao Tong University School of Medicine.

### Statistics

Statistical analyses were performed using either a Student’s *t*-test or one-way analysis of variance (ANOVA), according to the characteristics of the data. R software (3.6.2) and GraphPad Prism8 (GraphPad Software, San Diego, CA, United States) were used to perform data analysis and visualization.

## Results

### The Expression of PSMC5 Was Up-Regulated in Colorectal Cancer Tissue

**FIGURE 1:**
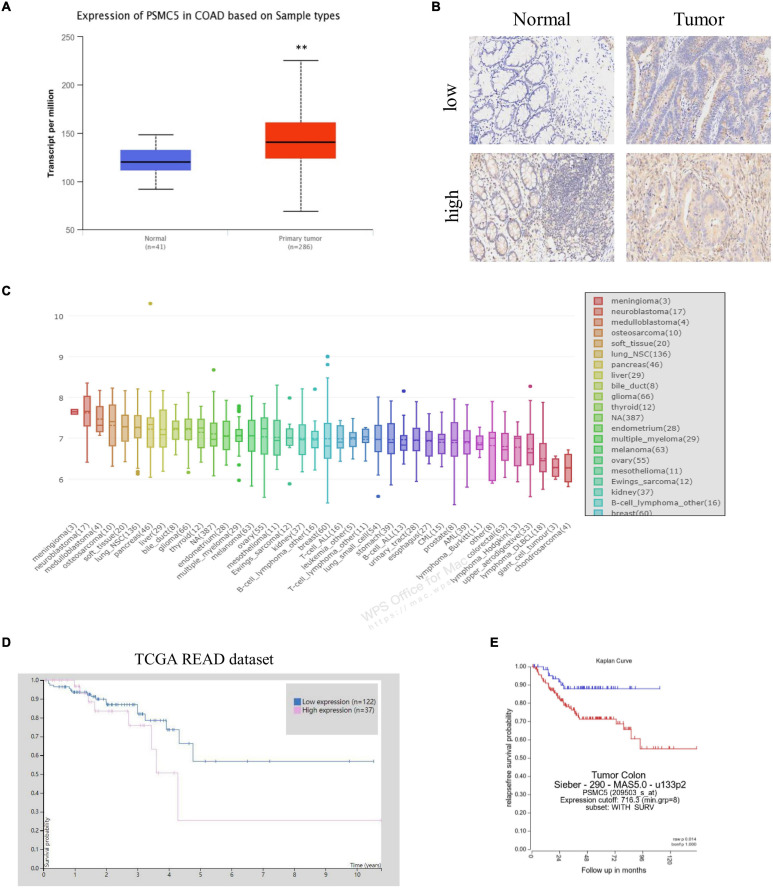
The expression of PSMC5 was significantly elevated in CRC tissues. **(A)** Expression level of PSMC5 in TCGA colon adenocarcinoma dataset. *p* values were calculated by Student’s *t*-test. **p < 0.01. **(B)** Representative IHC images of PSMC5 in CRC tissues and the paired normal tissues. **(C)** Expression of PSMC5 across multiple human cancer cell lines. **(D,E)** The Kaplan–Meier analysis of patients with high or low expression of PSMC5 in TCGA rectal adenocarcinoma (READ) and an independent colon cancer patient cohort [*p* = 0.01 **(D)** and *p* = 0.014 **(E)**, respectively]. CRC, colorectal cancer; TCGA, The Cancer Genome Atlas; and IHC, immunohistochemistry.

To demonstrate the potential effect of PSMC5 in CRC, we analyzed the expression pattern of PSMC5 in public CRC dataset. As shown, PSMC5 was significantly overexpressed in 286 COAD tissues than normal tissues (Figure 1A). Moreover, we enrolled 37 cases CRC patients in our institute and identified PSMC5 expression with immunohistochemistry staining of tumor and paired normal tissues. Consistently, higher PSMC5 expression was found in 62.2% (23/37) of cancer tissues. Meanwhile, in paired normal tissue, only 37.8% (14/37) cases showed a high PSMC5 level (*p* = 0.002, Figure 1B). Consistently, gain of PSMC5 was also observed in multiple human cancer cell lines including several CRC cell lines (Figure 1C). To further explore the role of PSMC5 in CRC, we next tested the clinical implication of PSMC5 by comparing the prognosis of CRC patients with the high or low level of PSMC5. The Kaplan–Meier analysis revealed that the low-expression group of CRC patients had a better long-term survival outcome (Figures 1D,E). Besides, PSMC5 was associated with more advanced tumor stage (Table 1). Taken together, the results above indicated that the expression of PSMC5 was elevated in CRC tissues compared with paired normal tissues, and high expression of PSMC5 indicated poorer prognosis of CRC patients.

**TABLE 1:** Relation of PSMC5 and clinical-pathology parameters.

Variables	Case (*N* = 37)	PSMC5 level		Statistical significance
		High (*n* = 23)	Low (*n* = 14)	
**Gender**				NS
Male	27	16	11	
Female	10	7	3	
**Age**				NS
≤60	16	7	9	
>60	21	16	5	
**Location**				NS
Ascending colon	9	6	3	
Transverse colon	11	5	6	
Descending colon	2	1	1	
Sigmoid colon and Rectum	15	11	4	
**Tumor size**				NS
≤5 cm	17	11	6	
>5 cm	20	12	8	
**TNM stage**				*p* < 0.01
I + II	17	6	11	
III + IV	20	17	3	

### PSMC5 Promoted the Proliferation and Invasion of Colorectal Cancer *in vitro* and *in vivo*

**FIGURE 2:**
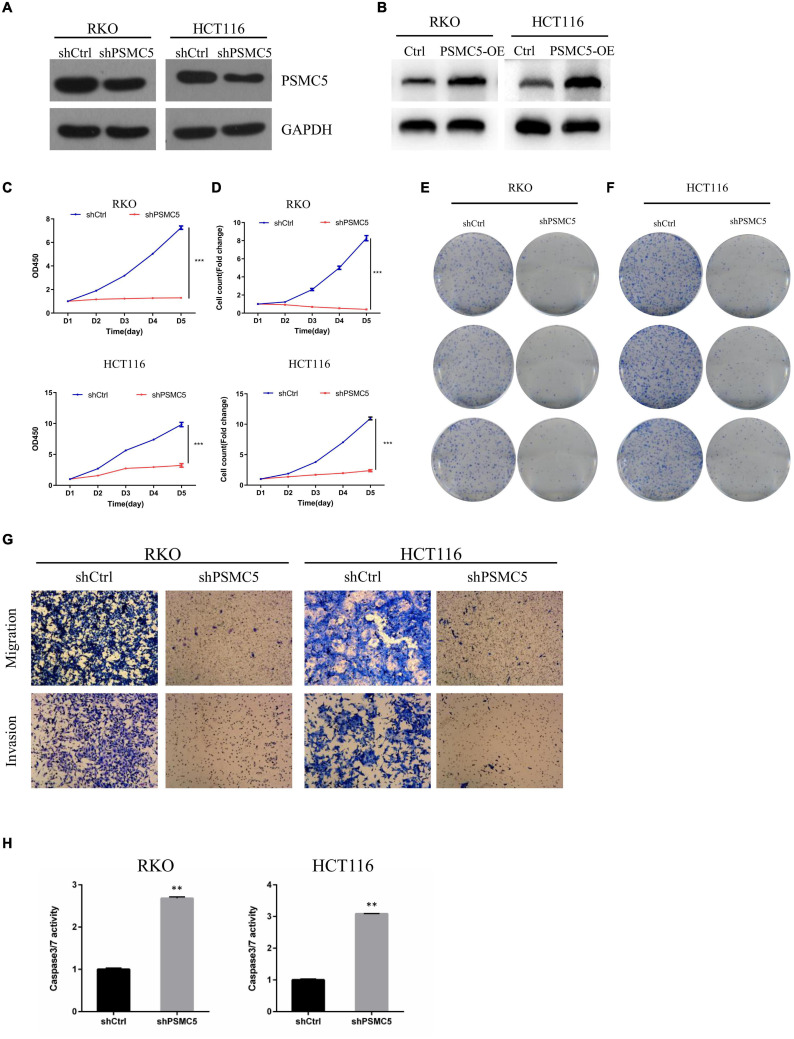
Loss of PSMC5 inhibits the ability of proliferation and invasion of CRC cells. **(A,B)** The effect of PSMC5 knockdown **(A)** and overexpression **(B)** was confirmed by Western blotting. **(C,D)** growth curve based on MTT assay **(C)** or cell count assay by the Celigo Image Cytometer **(D)**; one-way ANOVA test was used to determine the difference between two groups. ***p < 0.001. **(E,F)** Colony formation analysis of shPSMC5 and control cells in RKO **(E)** and HCT116 **(F)** cell lines. **(G)** Transwell assay showing the alteration of invasion cell numbers between shCtrl and shPSMC5 cells. **(H)** The level of caspase3/7 activity of shCtrl and shPSMC5 cells was examined and shown as mean ± sd. *p* values were calculated by Student’s *t*-test. *p* values were calculated by Student’s *t*-test. **p < 0.01. CRC, colorectal cancer.

**FIGURE 3:**
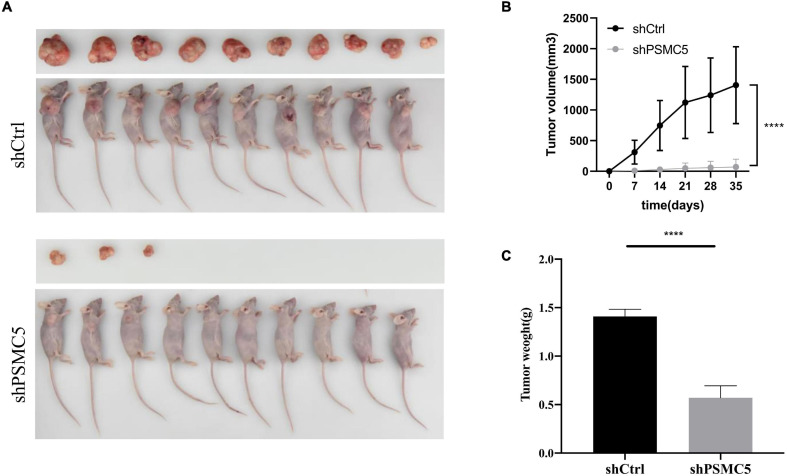
Effects of PSMC5 knockdown on tumor growth *in vivo*. **(A)** Images of tumors in nude mice. **(B)** Tumor volume measured per week in different groups. *p* values were calculated by one-way ANOVA test. ****p < 0.0001. **(C)** Tumor weight in shCtrl and shPSMC5 groups. *p* value was determined by Student’s *t*-test. ****p < 0.0001.

**FIGURE 4:**
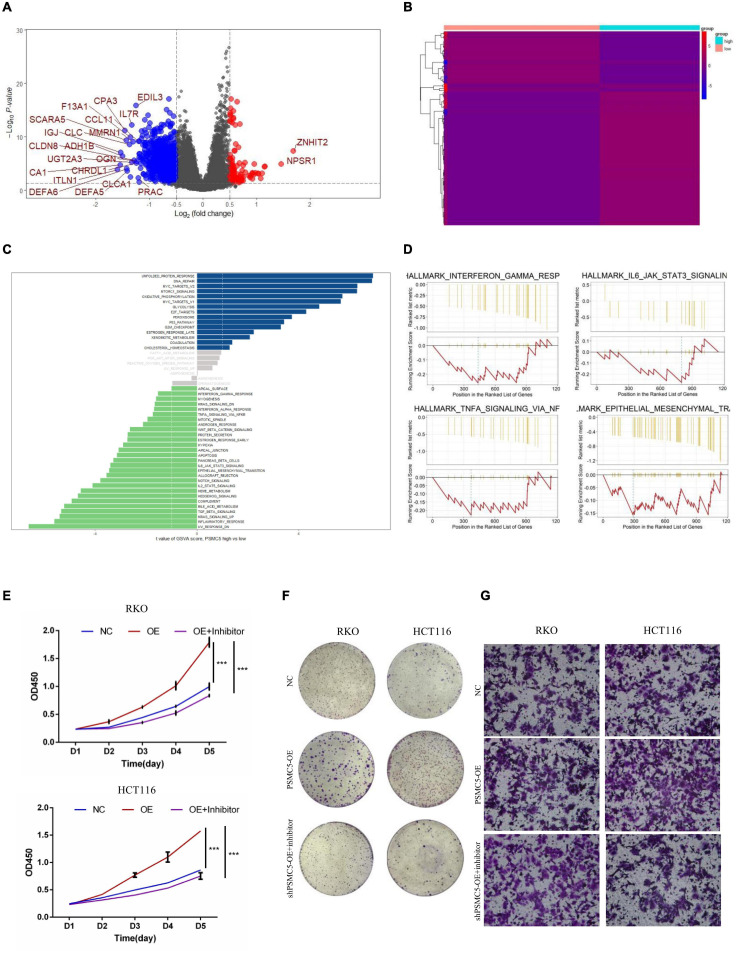
PSMC5 promoted proliferation and invasion of CRC cells by activating epithelial–mesenchymal transition. **(A,B)** Differentially expressed genes (DEGs) showed by volcano plot **(A)** or heatmap **(B)**. DEGs with log2FC > 1 are shown in the volcano plot. **(C,D)** PSMC5-related pathways were analyzed with Gene Set Variation Analysis (GSVA; **C**) and Gene Set Enrichment Analysis (GSEA; **D**) algorithm. **(E,F)** MTT assay **(E)** and colony formation assay **(F)** showing that the enhanced cell growth induced by PSMC5 could be dampened by EMT inhibitor. Difference across groups was determined by one-way ANOVA test. ***p < 0.001. **(G)** Transwell assay examined the invasive ability of three groups of CRC cells (control, PSMC5-OE, and PSMC-OE + EMT inhibitor). CRC, colorectal cancer; EMT, epithelial–mesenchymal transition.

To define the biological functions of PSMC5, we generated knockdown and overexpression cell models with PSMC5-targeting shRNA or pcDNA-PSMC5 plasmid in HCT116 and RKO CRC cells (Figures 2A,B and Supplementary Figures 1A,B). As expected, knockdown of PSMC5 significantly dampened the growth of CRC cells, which were reflected by the growth curve based on the cell count assay (Figure 2C) or MTT assay (Figure 2D). Moreover, colonies formed by shPSMC5 CRC cells were significantly decreased compared with those of the control group (Figures 2E,F and Supplementary Figures 1C,D). Next, we explored the impact of PSMC5 on metastatic potential of CRC cells. Transwell assays were conducted, and the results showed that the number of cells that passed through the chamber membrane in migratory and invasive assay was significantly decreased after PSMC5 was silenced (Figure 2G and Supplementary Figure 1E). These results indicated that loss of PSMC5 could apparently inhibit the proliferation and invasion of CRC cells. Additionally, the level of caspase-3/7 activity was examined to fully evaluate the alteration of cell viability of shPSMC5 cells. The results (Figure 2H) revealed that the caspase-3/7 activity was significantly elevated compared with control cells, implying that the loss of PSMC5 could promote apoptosis of CRC cells. By contrast, overexpression of PSMC5 led to the opposite changes in proliferation (Figures 4E,F and Supplementary Figure 1F) and invasion (Figure 4G and Supplementary Figure 1G) of HCT116 and RKO cells. Moreover, to validate the above observations, we established subcutaneous xenograft tumor model. shPSMC5 and control cells were subcutaneously injected into the flank of 4-week-old nude mice, and the tumor volume was measured weekly. Results revealed that tumor volume and average tumor weight were decreased in shPSMC5-injected nude mice (Figures 3A–C). Collectively, these results indicated that PSMC5 promotes proliferation and invasion of CRC cells *in vitro* and *in vivo*, indicating that targeting PSMC5 could be an effective approach to suppress growth and metastasis of CRC.

### PSMC5 Promoted Epithelial–Mesenchymal Transition of Colorectal Cancer Cells

**FIGURE 5:**
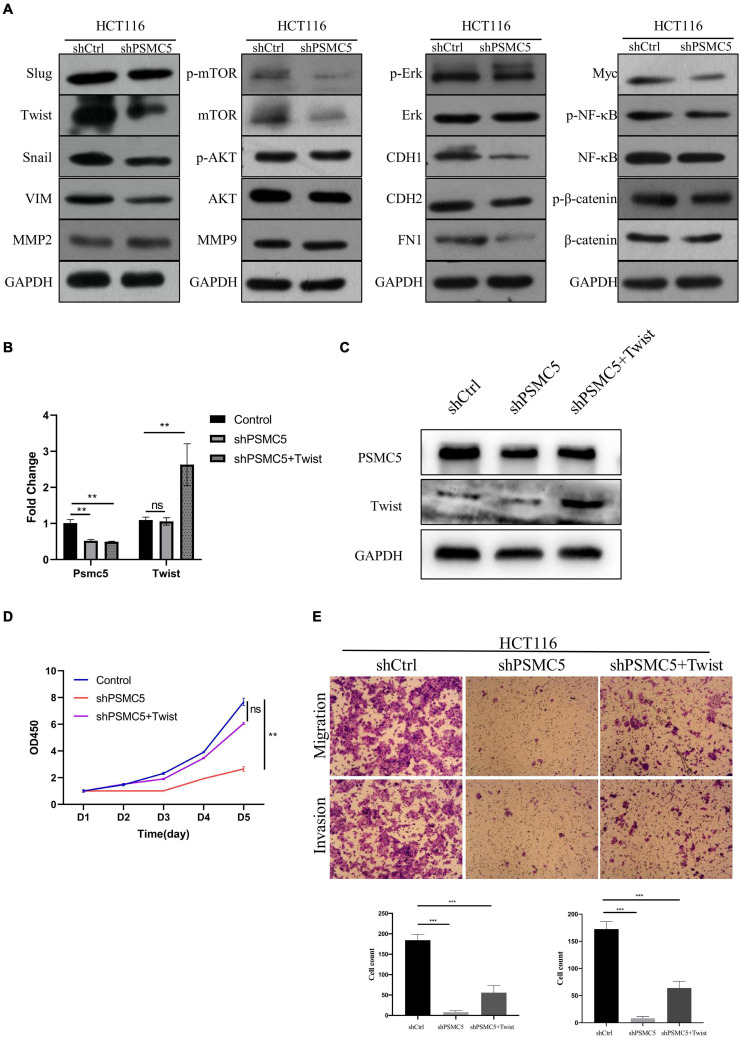
PSMC5 promoted epithelial–mesenchymal transition of CRC cells by upregulating *Twist*. **(A)** Western blotting panel containing key components of EMT was designed to screen the potential targets of PSMC5. Twist with the max fold change was selected for further research. **(B,C)** RT-PCR **(B)** and Western blotting **(C)** confirmed and validated co-transfection of *Psmc5* and *Twist*. Student’s *t*-test was used to measure the difference of target RNAs between control and transfected cells. **p < 0.01. **(D,E)** MTT assay **(D)** and transwell assay **(E)** showed that impaired cell growth and invasion could be rescued by ectopic expression of *Twist*. One-way ANOVA test was applied to calculate the difference of cell growth across three groups. Ns, not significant. **p < 0.01. CRC, colorectal cancer; EMT, epithelial–mesenchymal transition. ***p < 0.001.

To further investigate the molecular mechanisms of PSMC5 in CRC progression, we dichotomized RNA expression data from TCGA COAD and rectal adenocarcinoma into PSMC5-high and PSMC5-low groups. Differentially expressed genes (DEGs) were then analyzed with limma package, the result is shown in Figures 4A,B. We noted that several immune-related genes, such as IL7R, CCL11, and SCARA5, were tightly associated with PSMC5. Next, Gene Ontology analysis was carried out based on DEGs. As expected, PSMC5 was linked to multiple intracellular events such as chemokine binding, growth factor activity, and immunoglobulin binding (Supplementary Figures 2A–C), which might account for its potential interaction with EMT and tumor immune response. In addition, Gene Set Variation Analysis (GSVA) and Gene Set Enrichment Analysis (GSEA) were performed to further gain a comprehensive insight into detailed mechanisms. Consistent with the above results, PSMC5 was correlated with P53 pathway, mTOR pathway, G2M checkpoints, and EMT, which might be responsible for the promotion of tumor proliferation and suppression of tumor apoptosis. Moreover, several classical immune-related pathways, including interferon response, TGF-beta signaling, and IL6-JAK-STAT3 signaling, were shown to be involved in the process of PSMC5 regulating CRC cells (Figures 4C,D). To confirm bioinformatics results, PSMC5-overexpressed cells (PSMC5-OE) were cultured in medium containing EMT inhibitor (10 μm) for 24 h, and the alteration of proliferative and invasive abilities of CRC cells was measured. As shown, enhanced cell growth and invasion caused by ectopic PSMC5 expression were impaired by inhibition of EMT pathway (Figures 4E–G and Supplementary Figures 1F,G), which suggested that the biological function of PSMC5 in CRC was at least partly due to activation of EMT. Furthermore, Western blotting panel including the main EMT components were designed to screen the potential targets of PSMC5. Among all the dysregulated proteins, the expression of TWIST was drastically decreased with a maximum fold change after PSMC5 was knocked down (Figure 5A and Supplementary Figure 2D). To examine the hypothesis that PSMC5 might regulate Twist, we forced expression of Twist in shPSMC5 cells. The inhibited proliferation was partly rescued by expression of Twist (Figures 5B–D). Furthermore, the migratory and invasive abilities of shPSMC5 + Twist cells were elevated compared with shPSMC5 cells but were still inferior to those of shCtrl cells (Figure 5E). Taken together, PSMC5 might mediate the proliferation and metastasis of CRC through TWIST.

### PSMC5 Influenced the Infiltration Level of Immune Cells in the Tumor Microenvironment

**FIGURE 6:**
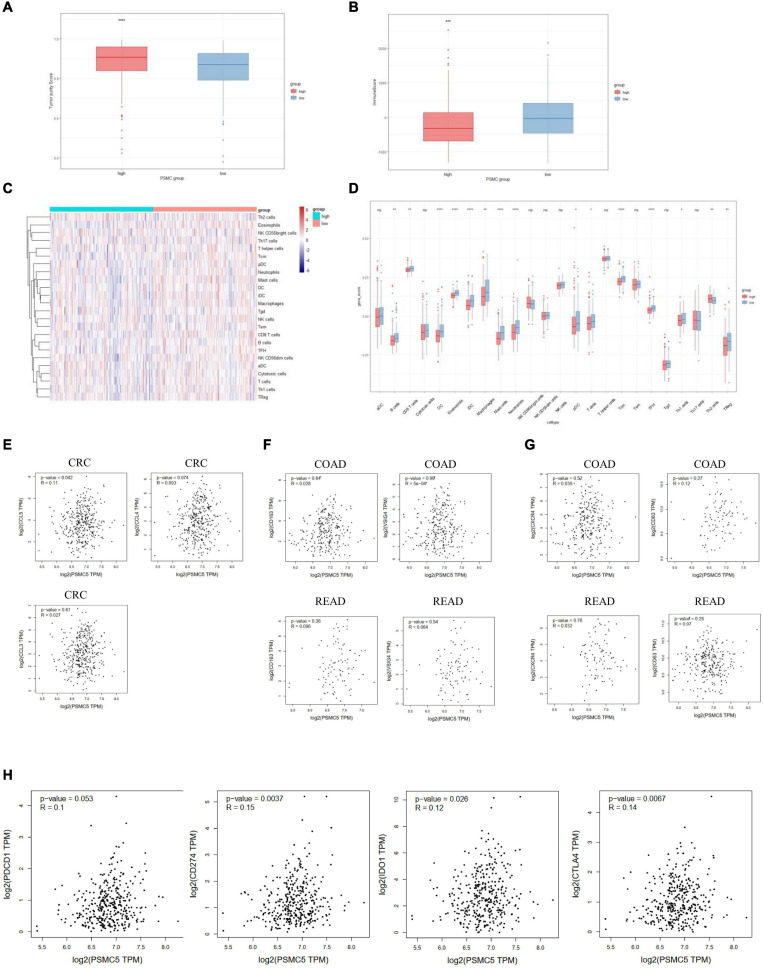
PSMC5 modulated several immune infiltrating cells in the tumor microenvironments. **(A,B)** Score of tumor purity **(A)** and immune cells **(B)** between PSMC-high and PSMC-low group. *p* value was generated from Wilcoxon’s test. **p < 0.01. **(C)** Heatmap showing the landscape of immune infiltrating cells of colorectal cancer. **(D)** Boxplot showing the difference of 24 kinds of immune cells between PSMC5-high and PSMC5-low groups. The significance of difference was calculated by Wilcoxon’s test. Ns, not significant. *p < 0.05, **p < 0.01, ***p < 0.001, and ****p < 0.0001. **(E–H)** Correlation analysis between PSMC5 and several chemokines (**E**; CXCL5 and CXCL8), M2 macrophages (**F**; CD163 and VSIG4), N2 neutrophils (**G**; CXCR4 and CD63), and immune modulators (**H**; PDCD1, CD274, IDO1, and CTLA-4). *R* and *p* value were determined by Pearson’s correlation test.

Recent studies showed that multiple types of tumor-associated immune cells configure a complicated microenvironment and influence tumor progression and treatment responses through variable mechanisms (Wu and Dai, 2017). As several immune-related pathways were reflected by GSVA and GSEA (Figures 4C,D), we next asked whether PSMC5 affected the infiltrating level of immune cells in TME and thereby modulated the immunotherapy response. Firstly, high PSMC5 expression was associated with lower tumor purity and higher immune score (Figures 6A,B), indicating that PSMC5 might regulate immune cell accumulation in the microenvironment of CRC. Next, we deconvoluted the CRC expression data with ssGSEA algorithm to evaluate the correlations between PSMC5 and immune infiltrating cells in TME. As shown, PSMC5 was negatively correlated with infiltrating levels of B cells, CD8 + T cells, macrophages, and neutrophils (Figures 6C,D). To figure out the mechanism by which PSMC5 attracts immune cells, we further measured the relationship of PSMC5 with several major chemokines. The results showed that PSMC5 was positively correlated with CCL3, CCL4, and CCL5 (Figure 6E), which were proved to be positive regulators of tumor-associated macrophage (TAM; Eissmann et al., 2019) and tumor-infiltrating neutrophil (TIN) abundance (Mukaida et al., 2020). TAM has two phenotypes, namely, antitumorigenic M1 macrophages and protumorigenic M2 macrophages. Numerous studies have demonstrated that M2 macrophage polarization acts as a major driving factor of CRC progression by activating several oncogenic pathways (Lan et al., 2019). Similarly, N1 and N2 neutrophils also display distinct functions in human cancer. We presumed that PSMC5 may increase the infiltration of M2 macrophages and N2 neutrophils. As expected, PSMC5 positively correlated with cell markers of M2 macrophages and N2 neutrophils (Figures 6F,G), which confirm our hypothesis. Moreover, we evaluated the relationship between PSMC5 and several immunostimulators, namely, PDCD1, CD274, CD80/CD86, LAG3, and CTLA-4. As expected, a higher PSMC5 level was significantly linked to expression of CD274 (PD-L1), CTLA-4, and IDO1 in CRC tumor cells (Figure 6H and Supplementary Figure 3A), indicating a potential role of inhibiting PSMC5 in immune checkpoint blockade (ICB) therapy. Similarly, PSMC5 was found to be either positively or negatively associated with treatment response of several cancers including melanoma, urothelial cancer, and non-small cell lung cancer (Supplementary Figure 3B). Taken together, the above results further confirmed that PSMC5 may regulate tumor immune response and could be regarded as a target to improve ICB therapy of several human cancers including CRC.

### Genetic and Epigenetic Alterations of PSMC5 in Colorectal Cancer

**FIGURE 7:**
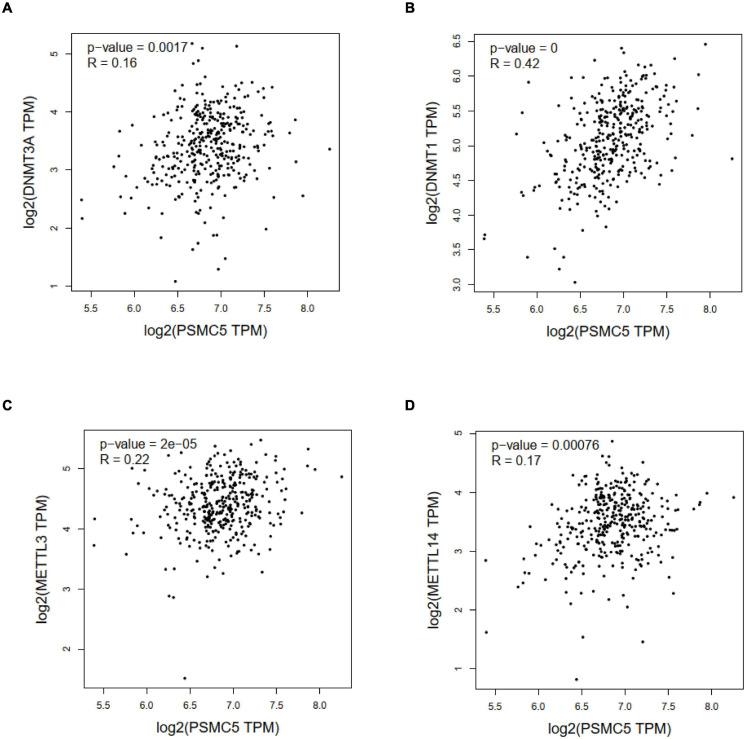
Correlation analysis between PSMC5 and DNA methyltransferase and m^6^A methyltransferases. Correlation between PSMC5 and DNMT1 **(A)**, DNMT3A **(B)**, METTL3 **(C)**, and METTL14 **(D)**.

Genetic and epigenetic changes play a critical role in regulating gene expression and cancer development. We next analyzed the copy number variation and DNA methylation level of *Psmc5* gene. Results showed that *PSMC5* mRNA expression was positively correlated with copy number (Supplementary Figure 3C), which suggested that gain or amplification of *Psmc5* gene might contribute to the upregulation of PSMC5 in CRC. By contrast, DNA methylation of *Psmc5* seemed to negatively regulate *PSMC5* mRNA level (Figures 7A,B). Recently, epigenetic modulation of RNA has emerged as an essential regulatory mechanism controlling gene expression and cell fate. *N*^6^-Adenosine methylation (m^6^A) is the most abundant internal post-transcriptional modification in eukaryotic cells. To explore whether m^6^A manipulated the expression level of *PSMC5*, we analyzed the potential m^6^A sites in *PSMC5* mRNA. According to the results generated from SRAMP online tools, we identified five m^6^A sites along the whole RNA sequence (Supplementary Figures 4A–F). Moreover, PSMC5 was significantly associated with METTL3 and METTL14, two major m^6^A methyltransferases, which validated our hypothesis that m^6^A plays an important role in the regulation of PSMC5 and CRC. Similar to DNA methylation, m^6^A is a dynamic process that needs to be recognized by “reader” protein to trigger downstream signaling. As expected, PSMC5 was significantly linked to members from several reader protein family, namely, IGF2BP2, IGF2BP3, hnRNPA2B1, hnRNPC, RBM15, and YTH family (Supplementary Figures 5A–E). Importantly, the interaction between PSMC5 and hnRNPC, YTHDC2, and RBM15 seemed to be very close (*p* = 7.7e-10, 1.1e-7, and 1.3e-4, respectively). As previously reported, these three readers participated in the regulation of RNA subcellular location, RNA stability, and RNA translation efficacy, reminding us that there were other mechanisms involved in the upregulation of PSMC5 in CRC. Collectively, these results indicated that epigenetic regulation was a key mechanism of PSMC5 in CRC.

## Discussion

In this study, we firstly reported that the expression of PSMC5 was higher in cancer tissues compared with normal tissues, and high expression of PSMC5 was associated with poor prognosis of CRC patients. Further investigations showed that knockdown of PSMC5 in CRC cell lines could suppress cell proliferation, colony formation, and metastasis but could promote cell apoptosis. Animal studies revealed that knockdown of PSMC5 inhibited tumor growth in nude mice. Mechanically, pathway analysis showed that PSMC5 regulated EMT of CRC cells, which was verified by the fact that decreased cell growth and invasion of shPSMC5 cells could be rescued by expression of *Twist*. Therefore, these results demonstrated that PSMC5 expression was a prognostic factor of CRC, and altered expression of PSMC5 in CRC cells had multiple functions on tumor cell behavior.

Epithelial cells can acquire infiltrating and metastasizing properties as a result of EMT (Mittal, 2018). Majority of tumors undertake EMT during tumor progression. The features during EMT include the loss of cell–cell junction, the downregulation of epithelial specific markers, the increase of mesenchymal markers, the acquisition of invasive phenotype, and apoptosis resistance (Thiery et al., 2009). Several key components including Snail, Slug, TWIST, and VIM significantly increased during the process of EMT. As shown in Figure 5, knockdown of PSMC5 resulted in downregulation of those EMT key factors, indicating that PSMC5 may regulate CRC phenotypes by modulating EMT process. As a key effector of EMT, overexpression of TWIST in cancer cells could lead to transition to mesenchymal phenotypes with enhanced migratory and invasive abilities of several types of cancer cells including hepatocellular carcinoma (HCC; Zou et al., 2015), ovarian cancer (Sun et al., 2016), and gastric cancer (Hsu et al., 2012). However, the role of TWIST has not been specified in CRC. In our study, forced expression of *Twist* in shPSMC5 cells could partly but significantly reduce the suppressed invasive and migratory abilities, indicating that PSMC5 regulates EMT through TWIST in CRC. PSMC5 is one of those six ATPases belonging to the 26S proteasome that can recognize and transfer ubiquitinated proteins for degradation (Jang et al., 2015; Yim et al., 2016). Moreover, there are evidences showing that PSMC5 was also involved in gene transcription. PSMC5 could mediate CDKN1A transcription by recruiting p53 to the promoter region of CDKN1A in response to ultraviolet radiation-induced DNA damage and could facilitate the damaging effects of radiation in radiation-responsive lung cancer cells. In this study, we uncovered that PSMC5 regulated EMT process by modulating TWIST. However, further investigations are needed to elucidate the molecular mechanism by which PSMC5 manipulates TWIST in CRC cells.

Malignant tumor is a complicated mixture of tumor cells, stromal tissues, and immune cells. Previous studies showed that infiltrated immune cells in TME, including TAMs, neutrophils, dendritic cells, and CD8 + T cells, were associated with the prognosis of CRC patients and response to ICB therapy (Tran Janco et al., 2015; Stanton and Disis, 2016; Fridman et al., 2017; Wu and Dai, 2017). In the current study, we found that PSMC5 regulated the abundance of several types of immune cells including neutrophils, macrophages, CD8 + T cells, and B cells in the peri-TME (Figure 6). Recently, the role of TINs and TAMs in cancers and ICB treatment were under extensive investigation (Ruffell and Coussens, 2015; Jackaman et al., 2017; Cai et al., 2019). However, either TAMs or TINs showed variable and even opposite functions in treatment response or prognosis of gastrointestinal cancer patients (Ostuni et al., 2015; Jackaman et al., 2017). The discrepancy of these studies reveals that there are different subgroups of tumor-infiltrating macrophages and TINs (Martínez et al., 2017; Giese et al., 2019; Vitale et al., 2019). Most immune cells in TME, including T cells, neutrophils, and macrophages, have been clustered into different subpopulations playing varied and even opposite roles in different disease contexts. TAM has two phenotypes, antitumorigenic M1 macrophages and protumorigenic M2 macrophages. Plenty of studies have demonstrated that the transition from M1- to M2-like macrophages is a key feature in the progress of cancer progression (Pandey, 2020). Similarly, N1 and N2 neutrophils also display distinct functions in cancer. N1 neutrophils possess potent antitumor activity by secreting immunostimulatory cytokines such as CCL3, CXCL9, and CXCL10, which facilitate recruitment and activation of CD8 + T cells (Fridlender and Albelda, 2012). In our study, PSMC5 is positively correlated with markers of protumorigenic M2 macrophage and N2 neutrophils, which indicated that PSMC5 might be involved in the state transition of TAMs and TINs. Moreover, expression of PSMC5 was shown to be related with response to ICB therapy in other cancers including lung cancer, melanoma, and urothelial cancer, which suggested that PSMC5 might be a potential marker or a target for ICB therapy in CRC patients.

At last, we explored the mechanisms governing the upregulation of PSMC5 in CRC. In addition to traditional genetic and epigenetic modulations, we surprisingly found that PSMC5 was significantly associated with key components of m^6^A (Figures 7C,D). Recently, m^6^A was proved to participate in the progression of multiple types of human cancers. METTL3 methylated and stabilized SOX2 mRNA and thereby promoted the stem cell property and invasion of CRC cells (Li et al., 2019). By contrast, METTL14 was reported as a tumor suppressor gene in several studies. The targets of METTL14 contained SOX4 (Chen et al., 2020a), miR-375 (Chen et al., 2020b), and lncRNA XIST (Yang et al., 2020). Reader protein YTHDF2 recognized m^6^A-marked SOX4 and XIST and induced the alteration of the stability and activity of downstream signaling (Chen et al., 2020a; Yang et al., 2020). Besides, METTL14 interacted with DGCR8 and affected the processing of pri-miR-375 (Chen et al., 2020b). In the current study, we demonstrated that PSMC5 mRNA sequence possessed several m^6^A sites and correlated with m^6^A transferases and reader proteins, indicating an important role of m^6^A in regulating PSMC5. As YTH family mainly influenced the stability and translation of mRNA (Wang et al., 2014; Shi et al., 2017), we presumed that the half-life of PSMC5 was prolonged and the translation efficacy of PSMC5 might be enhanced, resulting in the upregulation of PSMC5 in CRC cells. However, these theories needed to be verified in the future.

In summary, our study highlighted the clinical significance of PSMC5 in CRC and investigated the molecular mechanisms. Taken together, PSMC5 could regulate the proliferation and metastasis of CRC via modulating EMT and reshaping the TME. Knockdown of PSMC5 led to suppression of EMT and tumor metastasis, which may be a promising treatment strategy for CRC.

## Data Availability Statement

The raw data supporting the conclusions of this article will be made available by the authors, without undue reservation.

## Ethics Statement

The studies involving human participants were reviewed and approved by the Human Ethics Committee of Ruijin Hospital. The patients/participants provided their written informed consent to participate in this study. The animal study was reviewed and approved by Municipal Animal Experiment Center of Shanghai.

## Author Contributions

LZa, BF, and JM: conception and design. JS: development of methodology, writing, review, and revision of the manuscript. ZH, LH, LZh, and SZ: acquisition of data. ZH, LH, and LZh: analysis and interpretation of data. LZa and BF: administrative, technical, or material support. LZa, BF, and JM: study supervision. XY: analysis of TCGA public dataset. All authors read and approved the final manuscript for publication.

## Conflict of Interest

The authors declare that the research was conducted in the absence of any commercial or financial relationships that could be construed as a potential conflict of interest.
