# Supplementary material for: PSMC5 Promotes Proliferation and Metastasis of Colorectal Cancer by Activating Epithelial–Mesenchymal Transition Signaling and Modulating Immune Infiltrating Cells
Source: Front Cell Dev Biol. 2021 Jul 16;9:657917. doi: 10.3389/fcell.2021.657917 (PMC8323717; doi:10.3389/fcell.2021.657917)
Supplement: Supplementary file 1 [file Data_Sheet_1.docx]

**Supplementary Figure legends**

**Fig.S1** Statistical analysis of above results. (A, B) link to Fig.2A and Fig.2B. (C, D) link to Fig.2E and 2F. E links to Fig.2G. F links to Fig.4F. G links to Fig.4G.

**Fig.S2** GO analyisis of PSMC5 in colorectal cancer. A, B, and C indicated terms from Molecular Function (MF), Biological Process (BP), and cellular compartment (CC). D showed the statistical analysis of Fig.5A.

**Fig.S3** (A) Correlation analysis of PSMC5 and CD86, CD80 and LAG3. (B) PSMC5 was associated to treatment response of ICB therapy in multiple of cancers. The analysis was carried out using TISIDB online tools (http://cis.hku.hk/TISIDB/). (C-E) Evaluation of the influence of DNA methylation (C), copy number variation (D) and mutation (E) on PSMC5 expression level in TCGA CRC dataset.

**Fig.S4** (A) Prediction score of m6A status of *PSMC5* mRNA. (B-F) Diagram of potential m6A sites.

**Fig.S5** Correlation analysis between PSMC5 and m6A reader protein families namely WATP (A), IGF2BP family (B), hnRNP family (C), YTH family (D) and RBM15 (E).
